# Supplementary material for: Catalytic synthesis of n-butyl carboxylate with immobilized ionic liquid based on response surface methodology optimization
Source: R Soc Open Sci. 2019 Aug 14;6(8):190166. doi: 10.1098/rsos.190166 (PMC6731701; doi:10.1098/rsos.190166)
Supplement: The response surface diagrams of the interaction between the four factors [file rsos190166supp1.doc]

The response surface diagrams of the interaction between the four factors

Interactions exist among reaction time (A), molar ratio of acid to alcohol (B), reaction temperature (C) and catalyst dosage (D). Response surface analysis of the interaction between the four factors was performed, and response surface diagrams were drawn as shown in Figure S1- Figure S6


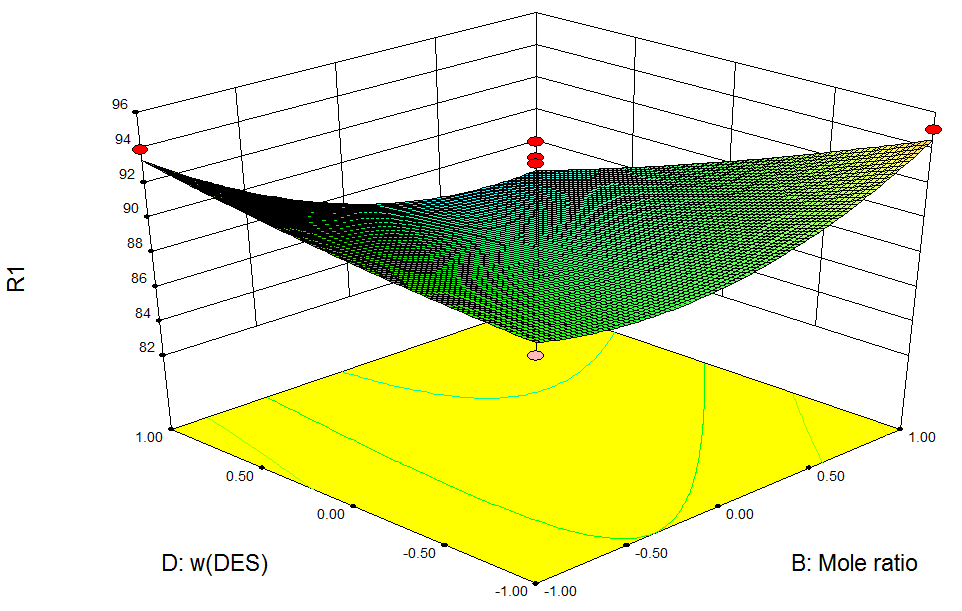


Figure S1 The 3D diagram of effect of B and D on yield


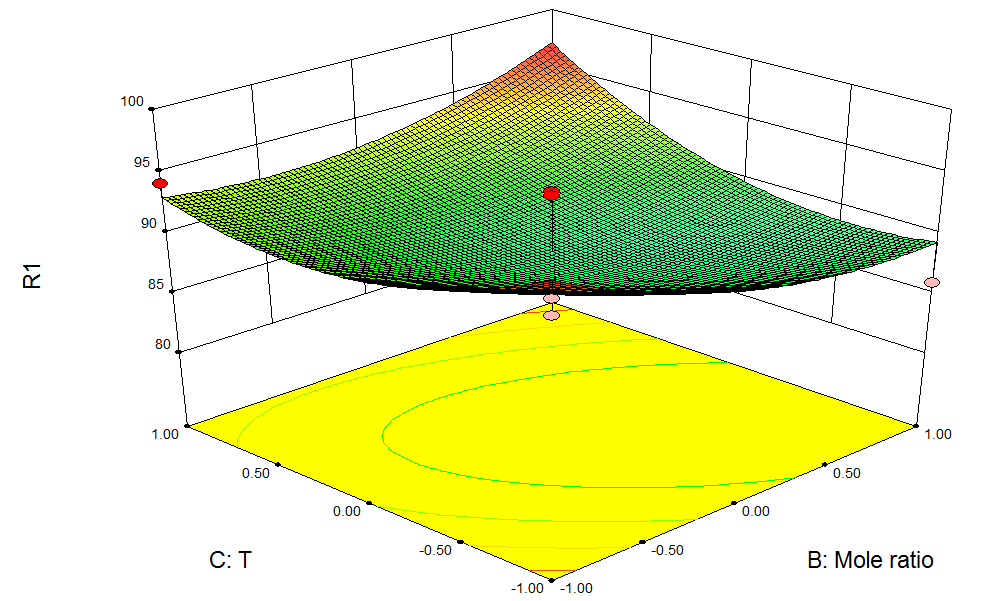


Figure S2 The 3D diagram of effect of B and C on yield


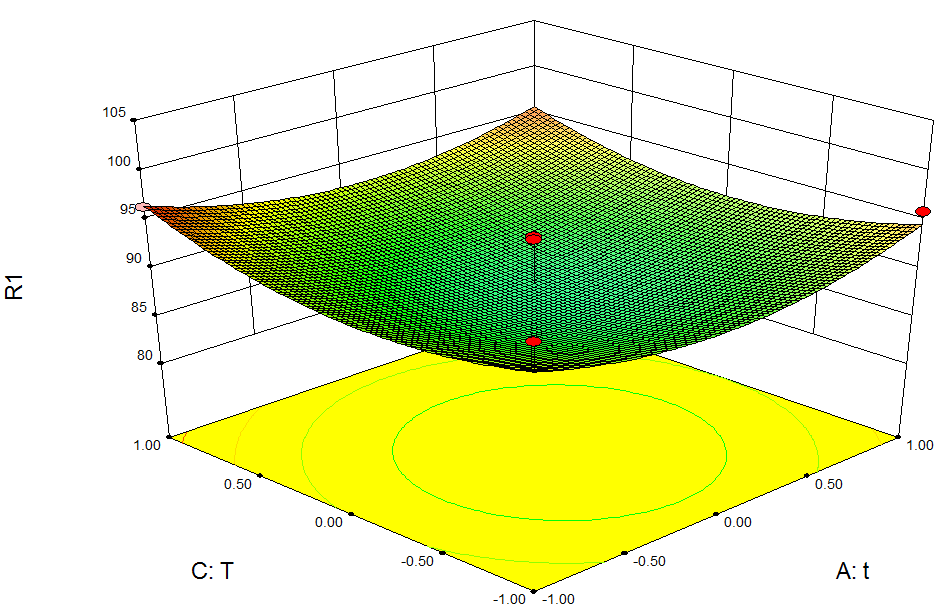


Figure S3 The 3D diagram of effect of A and C on yield


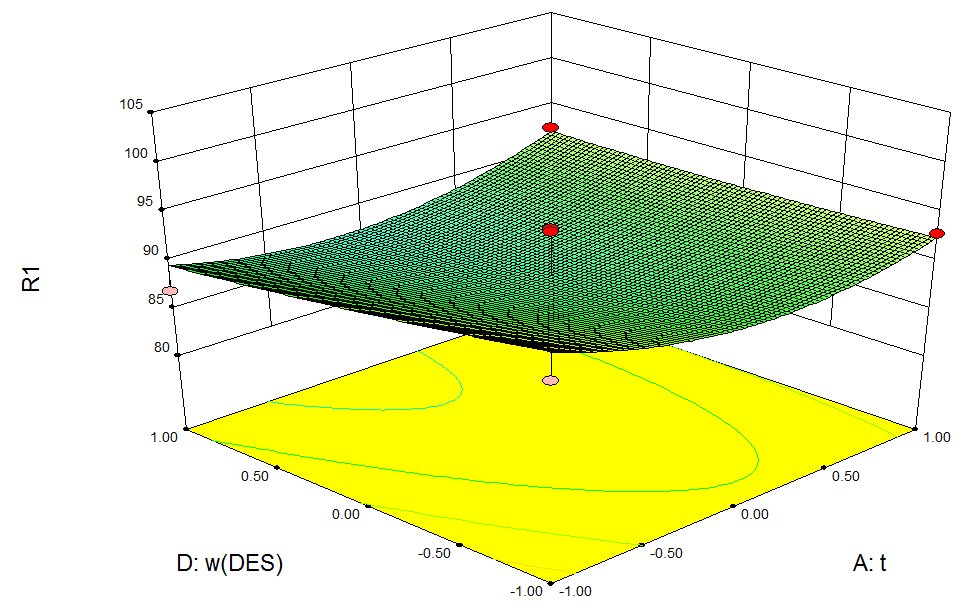


Figure S4 The 3D diagram of effect of A and D on yield


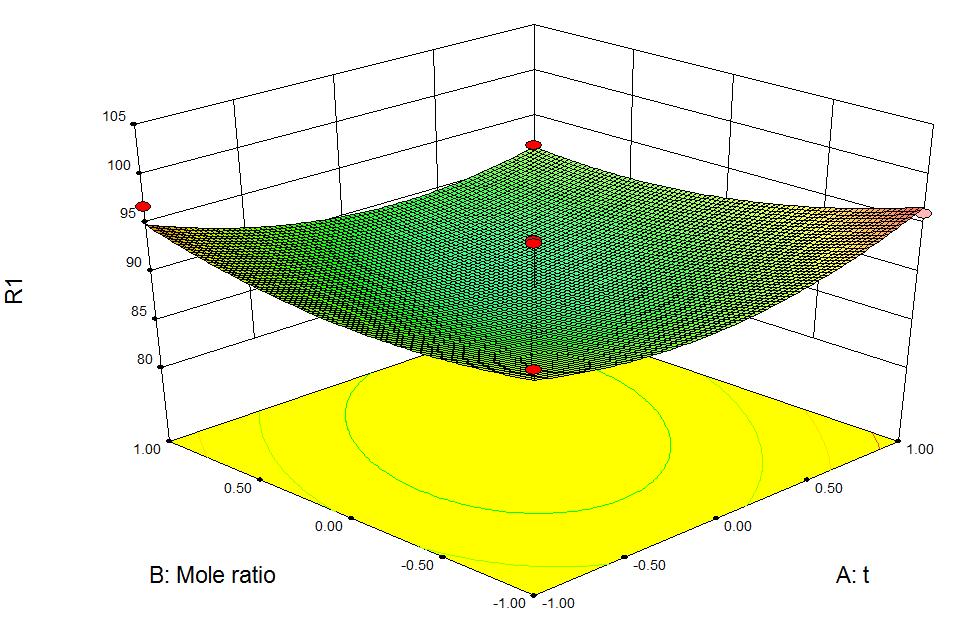


Figure S5 The 3D diagram of effect of A and B on yield


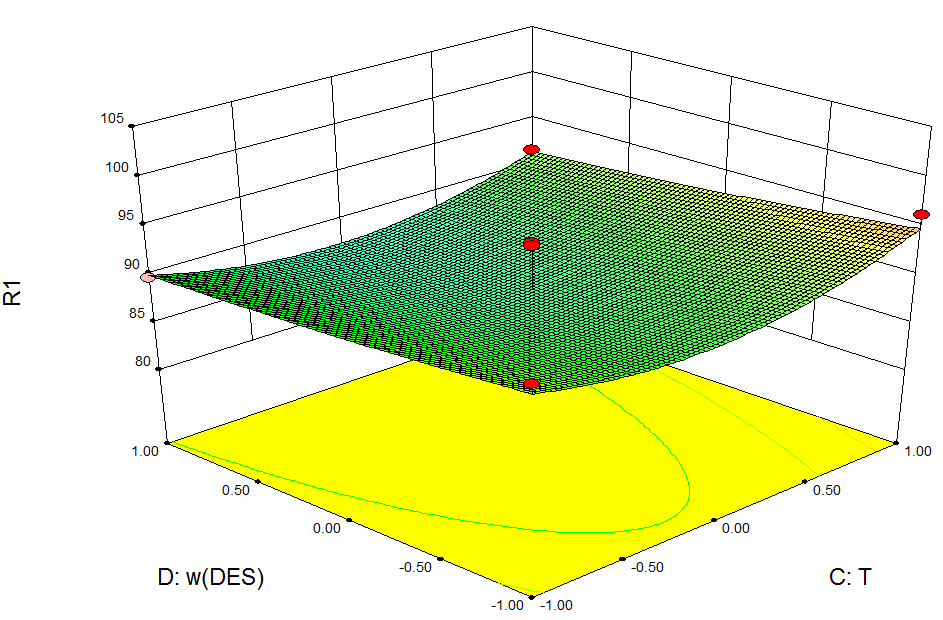


Figure S6 The 3D diagram of effect of C and D on yield
